# Live attenuated rubella vectors expressing SIV and HIV vaccine antigens replicate and elicit durable immune responses in rhesus macaques

**DOI:** 10.1186/1742-4690-10-99

**Published:** 2013-09-16

**Authors:** Konstantin Virnik, Max Hockenbury, Yisheng Ni, Joel Beren, George N Pavlakis, Barbara K Felber, Ira Berkower

**Affiliations:** 1 Lab of Immunoregulation, Division of Viral Products, Office of Vaccines, Center for Biologics, FDA, NIH Campus, Bethesda, MD 20892, USA; 2 Division of Veterinary Services Center for Biologics, Bethesda, MD 20892, USA; 3 Human Retrovirus Section, National Cancer Institute at Frederick, Bldg 535, Rm 210, Frederick, MD 21702, USA; 4 Human Retrovirus Pathogenesis Section, National Cancer Institute at Frederick, Bldg 535, Rm 209, Frederick, MD 21702, USA; 5 Office of Counter-Terrorism and Emergency Coordination (OCTEC), Center for Drug Evaluation and Research (CDER), FDA, 10001 New Hampshire Ave., Room 2131, Silver Spring, MD 20993, USA

**Keywords:** Live viral vector, Rubella vaccine strain RA27/3, Rhesus macaque, HIV MPER, SIV Gag, Highly immunogenic, Long lasting, Memory B cells

## Abstract

**Background:**

Live attenuated viruses are among our most potent and effective vaccines. For human immunodeficiency virus, however, a live attenuated strain could present substantial safety concerns. We have used the live attenuated rubella vaccine strain RA27/3 as a vector to express SIV and HIV vaccine antigens because its safety and immunogenicity have been demonstrated in millions of children. One dose protects for life against rubella infection. In previous studies, rubella vectors replicated to high titers in cell culture while stably expressing SIV and HIV antigens. Their viability *in vivo*, however, as well as immunogenicity and antibody persistence, were unknown.

**Results:**

This paper reports the first successful trial of rubella vectors in rhesus macaques, in combination with DNA vaccines in a prime and boost strategy. The vectors grew robustly *in vivo*, and the protein inserts were highly immunogenic. Antibody titers elicited by the SIV Gag vector were greater than or equal to those elicited by natural SIV infection. The antibodies were long lasting, and they were boosted by a second dose of replication-competent rubella vectors given six months later, indicating the induction of memory B cells.

**Conclusions:**

Rubella vectors can serve as a vaccine platform for safe delivery and expression of SIV and HIV antigens. By presenting these antigens in the context of an acute infection, at a high level and for a prolonged duration, these vectors can stimulate a strong and persistent immune response, including maturation of memory B cells. Rhesus macaques will provide an ideal animal model for demonstrating immunogenicity of novel vectors and protection against SIV or SHIV challenge.

## Background

Despite the urgent need for a safe and potent vaccine against HIV, efforts to produce a vaccine have been thwarted by antigenic variation, weak immunogenicity of critical epitopes, and short duration of the immune response to HIV vaccine antigens [1-3]. For other viruses, such as measles, mumps and rubella, similar immunogenicity problems were solved by developing live attenuated vaccine strains [4-6]. For HIV and SIV, live attenuated vaccines have been an attractive goal [7,8], but they may incur risks due to proviral integration and, in some cases, reversion to wild type virus [9,10]. Instead, live viral vectors with HIV vaccine inserts have been proposed [11-14] to combine the growth and immunogenicity of the vector with the antigenicity of the insert [15]. The live attenuated rubella vaccine strain RA27/3 is a promising viral vector, as its safety and immunogenicity have been established in clinical trials [5,16]. One dose of rubella vaccine elicits strong humoral and mucosal immunity and protects for life against rubella infection. By presenting HIV and SIV vaccine inserts as rubella antigens, live rubella vectors could enhance the immune response to these antigens.

We have recently identified two insertion sites in the rubella genome, where a foreign gene could be inserted without compromising rubella replication in cell culture [17-19]. The vector constructs were based on the rubella vaccine strain RA27/3 or on wild type rubella [20,21]. The first insertion site was located in the rubella non-structural region, where a permissive deletion between two Not I restriction sites [18,22] made room for an insertion at the same site. The insert was expressed as a fusion protein with nonstructural protein P150. This deletion/insertion strategy resulted in the first replicating rubella vectors capable of expressing zGFP, the HIV membrane proximal external region (MPER) determinant, and SIV Gag antigens [17,18]. However, since each insert was expressed as a fusion protein with P150, preservation of essential P150 functions could limit the size and composition of the insert.

The second insertion site was located in the structural region, between envelope proteins E2 and E1 [19]. This site uncoupled antigen expression from essential viral functions, and it accommodated larger and more complex antigens. At this site, insert expression was controlled by the strong subgenomic promoter, resulting in high-level expression for a longer duration. First generation vectors with a structural site insert retained the Not I deletion. These vectors grew to high titer in cell culture while expressing the insert at a high level [19]. Yet, their replication *in vivo* was compromised by the Not I deletion. New generation vectors, with structural site inserts and the Not I deletion restored, grew robustly *in vivo*, while expressing SIV and HIV vaccine antigens at high levels.

The rubella vaccine strain RA27/3 readily infects rhesus macaques [23]. The present study is the first to demonstrate the growth and immunogenicity of rubella vectors in macaques. The new generation rubella vectors infected all animals tested. They elicited a strong immune response to the SIV Gag insert, indicating the potency of vaccine antigens expressed at the structural insertion site. The antibodies were long lasting, and the animals responded strongly to a vector boost, indicating the induction of memory B cells. Rhesus macaques are also susceptible to SIV or Simian-Human immunodeficiency virus (SHIV) infection. This overlapping host range will provide an ideal animal model for immunizing with rubella vectors and testing protection against SIV or SHIV challenge [24,25].

## Results

### Vector constructs: replication and expression in cell culture

**Figure 1:**
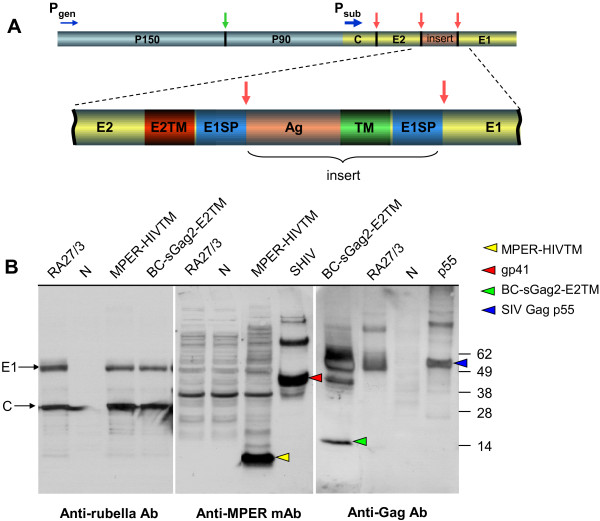
**Design of rubella vectors, their replication and expression of inserts at the structural site. (A)** The non-structural genes are located near the 5′-end of the genome (blue) and are controlled by the genomic promoter (P_gen_). The structural genes are located near the 3′ end (yellow) and are expressed as a structural polyprotein controlled by the strong subgenomic promoter (P_sub_). The structural insertion site is located between envelope glycoproteins E2 and E1. The antigenic insert (Ag, orange) is flanked on both ends by a transmembrane domain (red or green) and signal peptidase cleavage sequence E1SP (blue). The structural polyprotein is cleaved at three sites by signal peptidase (red arrows) to release mature structural proteins and the insert. **(B)** Replication of rubella vectors was demonstrated by Western blot of infected Vero cells with antibodies to rubella proteins E1 and C (left panel). Expression of the MPER-HIVTM insert was detected with monoclonal 2F5 (middle panel), while expression of the BC-sGag2-E2TM insert was detected with polyclonal HIV immune globulin (right panel). The control vaccine strain RA27/3 showed good growth (left panel), but no expression of either antigen (middle and right panels). The rubella-MPER vector replicated well (left panel), while expressing MPER-HIVTM as a 10 kDa band (middle panel, yellow arrowhead). The rubella-BC-sGag2 vector also replicated well (left panel) and expressed Gag as a 14 kDa band right panel, green arrowhead). Control lanes include AT-2 inactivated SHIV virions for gp41 (lane SHIV, red arrowhead), recombinant p55 SIV Gag protein (lane p55, blue arrowhead), and uninfected cells (lanes N).

The rubella genome is a 9.7 kb positive sense single-stranded RNA (Figure 1A). Genes at the 5′ end code for the nonstructural polyprotein, which is expressed under control of the genomic promoter and cleaved by viral protease (green arrow) to release mature nonstructural proteins P150 and P90 [21]. The 3′ genes code for the structural polyprotein, which is controlled by the strong subgenomic promoter. The polyprotein is normally cleaved at two sites by signal peptidase, to release mature structural proteins, capsid (33kDa), E2 (42–47 kDa) and E1 (58 kDa).

Three types of vectors were produced, with MPER or Gag sequences inserted into the RA27/3 rubella vaccine background. Type 1 vectors had an insert at the structural site and a compensatory deletion at the Not I site, as described previously [19]. Type 2 vectors had an insertion and deletion at the Not I site in the non-structural region of rubella [18]. The new type 3 vectors have an insertion at the structural site, but they have no deletion at the Not I site. Figure 1A shows the design of a typical type 3 rubella vector. The insertion site is located between envelope glycoproteins E2 and E1. The inserted sequences code for MPER of HIV-1 [18,19,26,27] or for an SIV Gag construct containing four T cell epitopes linked together (called BC-sGag2) [18,19,28,29]. Each antigenic insert (labeled Ag in Figure 1A) is preceded by the transmembrane domain of E2 (E2TM) and the signal peptidase site of E1 (E1SP), and it is followed by another transmembrane domain (TM) and E1SP peptidase site. Signal peptidase cleavage at three sites in the structural polyprotein (red arrows) would release the three rubella structural proteins plus the vaccine insert.

Vector replication in Vero cells was monitored by Western blot with antibodies to the rubella structural proteins C and E1 (Figure 1B, left panel). Rubella vectors expressing HIV MPER or SIV Gag antigen grew as well as the vaccine strain without an insert (left panel). Expression of the MPER-HIVTM insert was detected with anti-MPER monoclonal antibody 2F5 (Figure 1B, middle panel). The MPER insert was strongly expressed as a 10 kDa band (yellow arrowhead), which was absent in the empty rubella control (RA27/3, middle panel), and it was comparable to gp41 in the AT-2 inactivated virus control (SHIV, red arrowhead). Expression of the SIV Gag insert was detected by cross-reaction with HIV immune globulin (Figure 1B, right panel). The BC-sGag2 insert was expressed as a 14 kDa band (green arrowhead), which was absent in the empty rubella control (RA27/3, right panel) and was comparable to the control band for recombinant p55 Gag (lane p55, blue arrowhead). After 5 passages in cell culture, we expanded each vector to create viral stocks expressing MPER or SIV Gag inserts.

### Rubella vector stocks for *in vivo* studies

**Figure 2:**
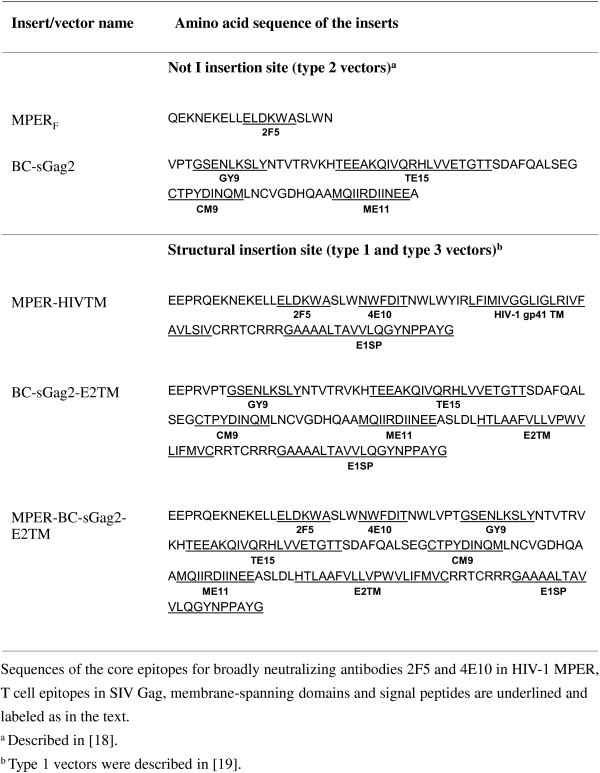
**Antigenic inserts used in this study.** Sequences of the core epitopes for broadly neutralizing antibodies 2F5 and 4E10 in HIV-1 MPER, T cell epitopes in SIV Gag, membrane-spanning domains and signal peptides are underlined and labeled as in the text. ^a^ Described in [18]. ^b^ Type 1 vectors were described in [19].

We produced and characterized six rubella vector stocks for monkey studies. We made two vectors of each type: one with an MPER insert and the other with a Gag insert. The MPER insert at the structural site consisted of the complete membrane proximal external region, followed by the HIV transmembrane domain and an E1SP signal sequence. At the nonstructural site, the MPER insert consisted of the 2F5 epitope alone, without the 4E10 epitope or transmembrane domain [18]. The SIV Gag insert at the structural site consisted of four T cell epitopes linked in tandem (called BCsGag2) followed by the E2TM domain of rubella and the E1SP signal peptide [19], while the Gag insert at the nonstructural site was BC-sGag2 alone. The sequences of all inserts are given in Figure 2. For each vector, we demonstrated insert expression by Western blot.

The titers of type 3 vector stocks are shown in Table 1. Viral RNA content was determined by quantitative RT-PCR. The viral titers were estimated by comparing their RNA concentration to that of a rubella reference sample of known PFU titer. Viral titers were 7.7 × 10^6^ PFU/ml, or greater, which is equivalent to about 1500 human doses per ml. This was 0.5 to 1 log greater than the viral titers reported previously for type 1 vectors [18,19], and it suggests that the new vectors replicate more robustly without a Not I deletion. Viral sequencing showed that the insert was stable and in reading frame after at least five passages. In addition, Western blot showed stable expression of the insert. The vector doses given to macaques, based on viral titer, were between two and ten times the typical human dose of rubella vaccine (about 5,000 PFU/dose).

**Table 1:** Titers of rubella vector type 3 stocks

**Viral vector (passage)**	**RNA copies/ml**	**Estimated titer, PFU/ml**
MPER-HIVTM (P_5_)	1.5 × 10^8^	1.3 × 10^7^
BC-sGag2-E2TM (P_5_)	8.5 × 10^7^	7.7 × 10^6^

### Rubella vector replication *in vivo*

**Figure 3:**
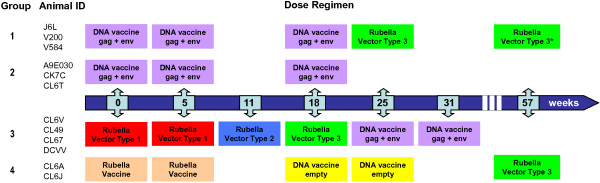
**Rhesus macaque immunogenicity protocol.** Macaques in group 1 received three doses of DNA vaccine, followed by a dose of rubella vectors at week 25 and a boost at week 57. Group 2 received DNA vaccine alone and were reserved for future studies. Group 3 received a series of three different rubella vectors, until the type 3 vectors gave a “take” in three out of three animals. They were boosted with two doses of DNA vaccine, starting at week 25. Group 4 control animals received rubella vaccine strain RA27/3 as an “empty” vector control, followed by two doses of control DNA plasmids, and a boost of rubella vectors at week 57.

The protocol for testing rubella vector replication and immunogenicity *in vivo* is shown in Figure 3. Macaques were immunized in groups of three animals, except for the control group of two animals. Group 1 received three priming doses of DNA vaccine, followed by a boost with rubella vectors. Group 2 received three doses of DNA vaccine and were reserved for future study. Group 3 received a series of rubella vectors first, followed by two doses of DNA vaccine. Group 4 controls received the rubella vaccine strain twice (with no insert), followed by empty DNA vaccine. Rubella vectors were given pairwise: one vector expressed MPER and the other expressed BC-sGag2, and both inserts were at the same insertion site. The first vector was given at a dose of 10,000 PFU initially, followed by 30,000 PFU for the second dose. The second and third vectors were given at 50,000 PFU per dose (approximately 10 human doses). At week 57, macaques in groups 1 and 4 were given a boost with type 3 vectors to determine feasibility of boosting.

The DNA vaccine consisted of SIV gag and HIV clade B env at the first dose, clade C at the second dose, and both clades for the third dose [30]. Mouth swabs were taken before each dose of live rubella vectors and one and two weeks after the dose to detect viral RNA by RT-PCR. Blood samples were taken before each dose and one, two and six weeks after immunization to analyze the immune response to rubella proteins and to each insert.

**Figure 4:**
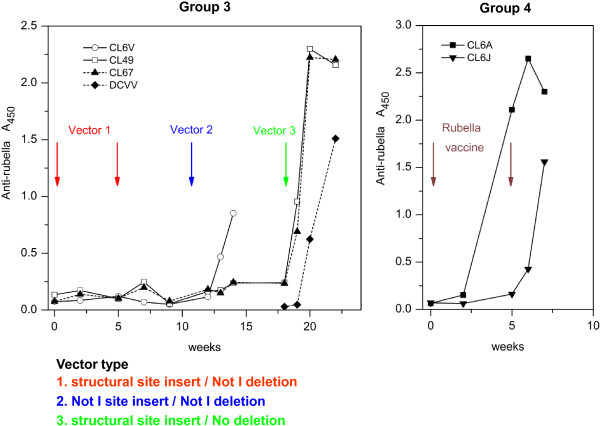
**Immune response to rubella antigens as a measure of a vaccine “take”.** Group 3 macaques were inoculated with a series of three types of rubella vectors bearing inserts at different insertion sites (left panel). These were compared with group 4 macaques, which received the same live attenuated rubella vaccine strain but without an insert (right panel). A successful vaccine “take” was detected by the appearance of anti-rubella antibodies, as measured by ELISA. Vector type 1, with a structural site insert and Not I deletion (red), elicited no detectable antibodies after two vaccinations. Vector type 2, with insertion and deletion at the Not I site (blue), gave a “take” in one of three animals. Vector type 3, with a structural site insert and no deletion (green), infected all three macaques after a single dose.

According to the protocol, group 3 macaques were the first ones to receive live rubella vectors. We followed their immune response to rubella structural proteins as an indicator of vector replication *in vivo* (Figure 4). The first two doses of type 1 vectors failed to elicit antibodies to rubella (left panel). The next dose of type 2 rubella vectors gave a partial “take” in one out of three animals (CL6V). At this point, we replaced the seropositive animal with a naïve macaque (DCVV), in order to have three rubella seronegative animals for the next injection. The next dose consisted of type 3 vectors and was given at week 18. All three animals promptly seroconverted, indicating a vaccine “take”. Anti-rubella titers peaked after 2 to 4 weeks (left panel, CL49, CL67, and DCVV). The magnitude and kinetics of the anti-rubella response elicited by live attenuated rubella vectors (left panel) were similar to control macaques in group 4 that received live attenuated rubella vaccine without an insert (Figure 4, right panel). Type 3 rubella vectors retained potency for rubella antigens while expressing vaccine antigens at the structural site.

**Figure 5:**
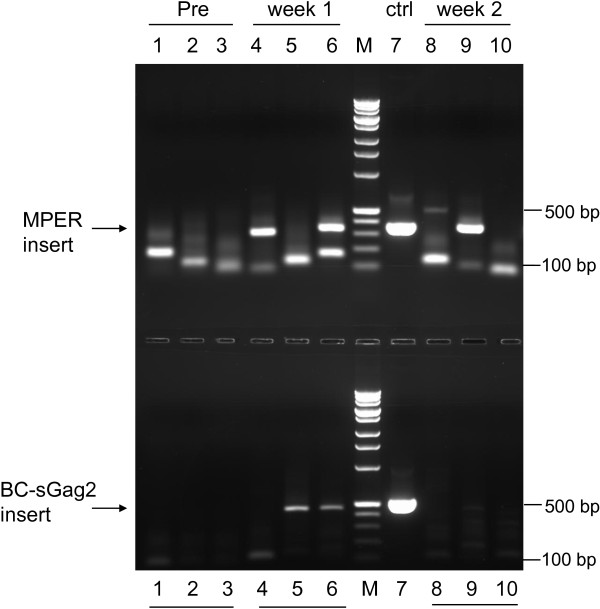
**Detection of systemic infection with rubella vectors by RT-PCR.** Group 1 macaques were immunized with live rubella vectors given IM in the quadriceps, and gingival mouth swabs were collected at 0, 1, and 2 weeks after immunization. Viral RNA containing the MPER insert was amplified by RT-PCR, and the products were resolved in 2% agarose gel. Lane 7 shows the PCR band at 347 bp for a plasmid with the same MPER insert as in the viral vector (upper panel). No virus was detected in pre-injection samples (lanes 1–3, representing J6L, V200, and V584). One week post infection, two of three animals were positive for virus (lanes 4 and 6). By week 2, both of these were resolved (lanes 8, 10), and the third animal was positive (lane 9). Similar results are shown for RT-PCR of the BC-sGag2 insert (lower panel), with a positive band at 494 bp for the plasmid control (lane 7). All animals were negative at the start (lanes 1–3), two of three were positive at week 1 (lanes 5 and 6), and both had cleared by week 2 (lanes 9 and 10). We detected no signal for BC-sGag2 from one of the macaques (J6L) at either time point.

By week 25, group 1 macaques had been primed with three doses of DNA vaccine, and they were ready for a rubella boost. They were given a single dose of the same type 3 vectors that replicated in group 3. As before, all three macaques promptly became infected with rubella vectors. The vectors were injected in the quadriceps muscle, and vector proliferation and dissemination were tested a week later by RT-PCR of mouth swabs. Pre-immunization swabs were all negative for rubella vectors bearing the MPER insert (Figure 5 upper panel, lanes 1–3). A control DNA plasmid containing the identical MPER insert (lane 7) gave a PCR band at 347 bp. One week after infection, two out of three macaques were positive for this band (lanes 4 and 6), and the third macaque became positive after two weeks (lane 9). Similarly, there was no pre-immunization RT-PCR signal for SIV Gag (lower panel, lanes 1–3). The plasmid control gave a band at 494 bp (lane 7). Two macaques became positive for this band after one week (lower panel, lanes 5 and 6), although the third macaque gave no signal for Gag at either time point (lanes 4 and 8). The results showed three patterns: one macaque was positive for both vectors at the same time and cleared the virus by week 2 after vaccination (lanes 6 and 10 in both panels); one macaque was positive for the SIV Gag vector first, and then for the MPER vector the following week (lanes 5 and 9, both panels); and one macaque was positive for the MPER vector, but never gave a signal for SIV Gag (lanes 4 and 8, both panels). The results indicate that two rubella vectors can grow side by side in the same host. Since the vectors were given in the quadriceps muscle and detected in mouth swabs one or two weeks later, this result indicates systemic infection by rubella vectors in all three animals. Failure to detect a PCR signal for the rubella-Gag vector in one macaque is not surprising for this method, which depends on the sensitive detection of low virus titers in the mouth [16,31].

### Immunogenicity of Gag vector inserts

**Figure 6:**
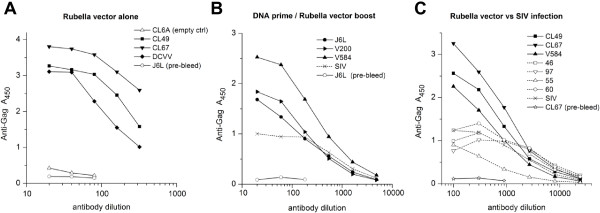
**The immune response to the SIV Gag insert was measured by ELISA on plates coated with recombinant p55 SIV Gag protein. (A)** Group 3 macaques, which received rubella vectors alone (live vector + 7 weeks), or **(B)** group 1 macaques, which received DNA prime and rubella vector boost (live vector + 4 weeks), produced high-titer anti-Gag antibodies in six out of six animals, as compared to the positive control from a pool of SIV infected macaques. The rubella vaccine control (CL6A empty in panel **A**) elicited no antibodies. **(C)** Anti-Gag antibody titers in three immunized macaques from groups 1 and 3 (four weeks after live vector, solid lines) were compared to a panel of four macaques infected 32 weeks earlier with SIV and a pool of infected macaques (dotted lines). Anti-Gag titers elicited by vector immunization were comparable to the antibodies elicited by SIV infection.

The antibody response to the SIV Gag insert was measured by ELISA assay on plates coated with recombinant SIV p55 Gag protein (Figure 6). High-titered antibodies to SIV Gag were elicited in all six macaques in group 3 (Figure 6A) and group 1 (Figure 6B). The antibodies were not observed in pre-bleeds or in control animals that were immunized with rubella vaccine lacking an insert (macaque CL6A), indicating that the antibodies were made in response to the insert. The anti-Gag titers from three vector-immune macaques in group 1 were greater than or equal to a pool of positive sera from six SIVmac251 infected macaques (Figure 6B). Unlike SIV infection, which exposes macaques to the full-length Gag protein, the BC-sGag2 vector covered only 20% of the Gag sequence, yet it elicited antibody titers comparable to infection.

We compared three vaccinated macaques (two from group 3 and one from group 1) with a panel of five macaques infected with SIVmac251 (Figure 6C). In each case, immunization with Gag vectors elicited anti-Gag antibodies with higher maximum ODs by ELISA and similar endpoint titers as those induced by natural SIV infection. This result indicates the potency of vaccine antigens expressed at the structural insertion site of live rubella vectors.

**Figure 7:**
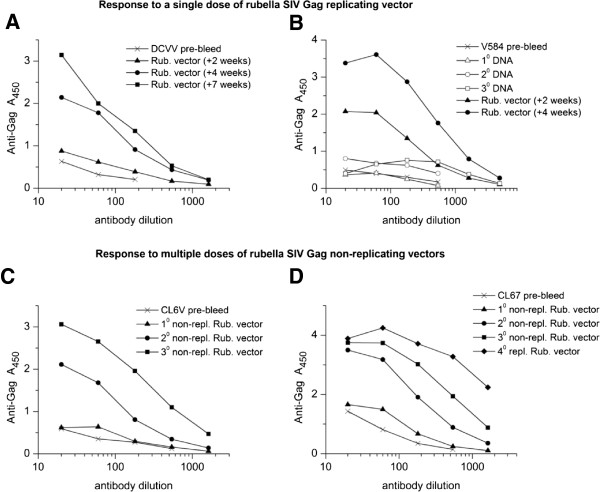
**Antibody response to replicating (A and B) and non-replicating (C and D) rubella vectors. (A)** Macaque DCVV received a single dose of replicating vectors. Anti-Gag antibodies were detectable 2 weeks post immunization and they rose progressively by weeks 4 to 7. **(B)** Macaque V584 was primed three times with DNA vaccine, which elicited a good antibody response, and these titers rose substantially between 2 and 4 weeks after a single dose of live vectors. **(C, D)** Macaques CL6V and CL67 received three doses of non-replicating vectors. Antibodies were barely detectable after the first dose (5 weeks post immunization), but titers rose progressively after the second (4 weeks post immunization) and third doses (2 weeks post immunization), eventually reaching the same levels as a single dose of live vectors.

The kinetics of the response to SIV Gag are shown for individual macaques (Figure 7) to illustrate the difference between live and non-replicating rubella vectors. Two animals, DCVV (group 3) and V584 (group 1), demonstrate the sustained response elicited by a single dose of live rubella vectors (Figure 7A and B). For DCVV, anti-Gag antibodies rose between 2 and 4 weeks and reached a peak 7 weeks post immunization (Figure 7A). For macaque V584, which received three priming doses of DNA vaccine, a good anti-Gag titer was elicited by the DNA vaccine. This was followed by an even stronger anti-Gag response to the live vector that increased steadily between 2 and 4 weeks post immunization (Figure 7B). The specificity of these antibodies for SIV Gag was demonstrated by Western blot (19).

In contrast, macaques CL6V (Figure 7C) and CL67 (Figure 7D) were initially given two doses of non-replicating type 1 vectors that expressed the same inserts at the same site as the replicating vectors, followed by a third dose with a type 2 vector. Both macaques made a minimal response to the first dose of non-replicating vectors. However, two or three doses of non-replicating vectors could achieve the same antibody titers as a single dose of the same insert in a live vector.

### Immunogenicity of MPER vector inserts

**Figure 8:**
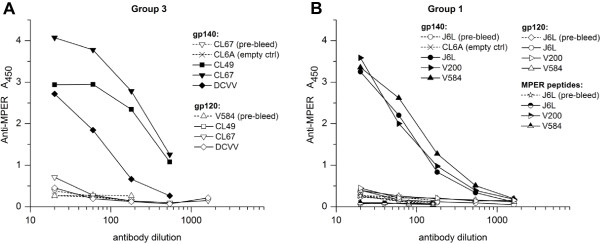
**The immune response to the MPER HIV-1 insert.** Five out of six macaques in group 3 **(A)** and in group 1 **(B)** produced high-titered antibodies in response to the MPER insert. One animal showed a moderate response. The sera were tested four to seven weeks after immunization with replicating vectors in group 3 macaques and four weeks after live rubella vectors in group 1. The empty rubella vaccine control (CL6A) elicited no antibodies, indicating specificity for the insert. No binding to gp120 was observed in any serum, indicating antibody specificity for the MPER insert. However, the group 1 antibodies did not bind monomeric MPER peptides corresponding to the 2F5 and 4E10 epitopes, and similar results were obtained with group 3 antibodies.

The antibody response to the HIV MPER insert was measured by ELISA on plates coated with gp140 SOSIP trimers (Figure 8). High-titered antibodies to MPER were elicited in five out of six macaques in group 3 (Figure 8A) and group 1 (Figure 8B), while one animal showed a moderate response. The antibodies were not observed in pre-bleeds or in control animals that received rubella vaccine without an insert (CL6A control). The antibodies were specific for the external domain of gp41, since they bound gp140 but not gp120. The antibodies may be specific for MPER determinants in gp140 trimers: they bound SOSIP gp140, which are enriched in trimers [32], but they did not bind monomeric peptides containing the 2F5 and 4E10 epitopes (Figure 8B).

### Antibody persistence

**Figure 9:**
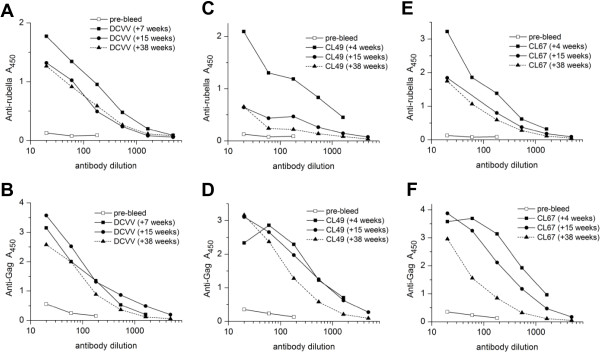
**Persistence of anti-rubella and anti-Gag antibodies elicited by rubella vectors in group 3 macaques.** The midpoint titers of anti-rubella antibodies **(A, C, E)** and anti-Gag antibodies **(B, D, F)** were measured for each animal between weeks 22 to 56 of the study, corresponding to weeks 4 to 38 after rubella immunization. Both antibodies peaked by 4 to 7 weeks after immunization. For DCVV, the decline in rubella antibodies and anti-Gag antibodies were both less than 3-fold over 34 weeks. For CL49, anti-Gag antibodies declined about 3-fold, while the decline in rubella antibodies was much greater. For CL67, anti-Gag antibodies declined about 13 -fold, which was greater than the decline in anti-rubella titers.

We measured the persistence of anti-Gag antibodies in group 3 macaques for nine months after immunization. We compared this to the persistence of anti-rubella antibodies in the same animals (Figure 9). Anti-rubella titers in all three macaques (Figure 9A, C and E) peaked 4 to 7 weeks after immunization. Based on midpoint titers, the antibodies declined about 3-fold by 15 weeks post immunization for DCVV and CL67 and then remained constant until 38 weeks. The decline was greater for CL49, about 9-fold by 15 weeks and another 3-fold by 38 weeks.

We measured anti-Gag antibodies in the same macaques at the same time points (Figure 9B, D, and F). Anti-Gag antibodies peaked 4 to 7 weeks post immunization (week 22 to 25 of the study) and then declined 3-fold or less by 15 weeks. By 38 weeks post immunization (study week 56), they declined another 3-fold or less in two macaques (DCVV and CL49) and 5-fold in the other (CL67). In two macaques (DCVV and CL49), the durability of anti-Gag titers was greater than or equal to anti-rubella titers, and in one case (CL67) anti-Gag titers were less durable over a 9 month period. This suggests that the persistence of anti-Gag antibodies could be comparable to anti-rubella antibodies, which are known to be long-lasting [16]. The durability of anti-Gag antibodies between 7 and 15 weeks after vaccination may be due, in part, to two boosts of DNA vaccine given during this time. However, this would not account for the continued stability observed during weeks 15 to 38 post vaccination, when DNA vaccine was not given.

**Figure 10:**
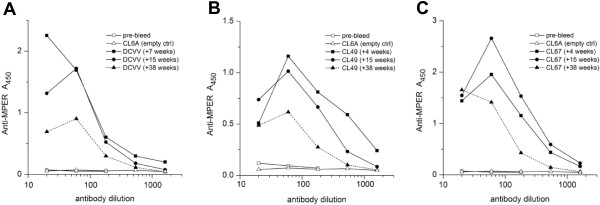
**Persistence of anti-MPER antibodies elicited by rubella vectors in group 3 macaques.** Anti-MPER antibody titers were measured for group 3 animals, DCVV **(A)**, CL49 **(B)** and CL67 **(C)**, between weeks 4 and 38 after live vector immunization. The same sera were tested as in Figure 9. The antibodies were near peak titers at 4 to 7 weeks post immunization. The titers declined 2 to 3-fold by 38 weeks, based on half-maximum binding, and this was nearly the same as anti-rubella antibodies in the same animals.

We measured the persistence of anti-MPER antibodies in the same group 3 macaques at the same time points (Figure 10A, B, and C). Anti-MPER antibodies reached high titers by 4 to 7 weeks post immunization. The titers changed very little at 15 weeks, when all three animals were within two-fold of the peak titer. By 9 months, the antibodies declined 2 to 3-fold, as judged by half-maximum titer. The durability of anti-MPER antibodies was comparable to anti-rubella antibodies, at least for the first nine months post immunization.

### Response to a rubella vector boost

We waited up to a year for rubella antibodies to decline to a level where we could boost with rubella vectors bearing new antigens. For group 4 macaques, these antibodies declined about 2.5-fold after 6 months, and then remained constant or rose slightly by 1 year. After one year (group 4) or 6 months (group 1), we boosted the macaques with rubella vectors expressing MPER and SIV Gag antigens (week 57 of the study). All five animals showed a prompt rise in antibodies to rubella (data not shown).

**Figure 11:**
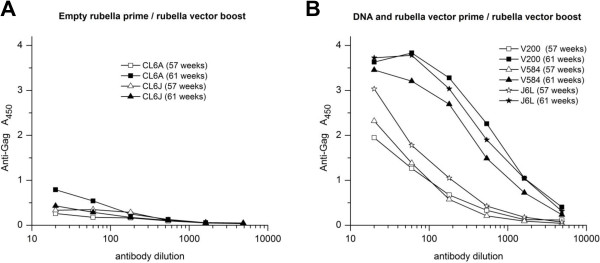
**Anti-Gag response to a live vector boost. (A)** Group 4 macaques were primed with the rubella vaccine strain at 0 and 5 weeks. They were boosted at week 57 with rubella vectors expressing BC-sGag2-E2TM and HIV MPER. **(B)** Group 1 macaques were primed three times with DNA vaccine, followed by a live BC-sGag2 vector at week 25. They were boosted at week 57 with novel vectors expressing an MPER–BC-sGag2-E2TM fusion protein and the entire p27 Gag protein. Animals primed with empty rubella vaccine had no antibodies to Gag and failed to respond to the BC-sGag2 boost. Animals primed with gag DNA and rubella expressing BC-sGag2 had persistent antibodies at week 57 (open symbols), and they responded strongly to the boost (solid symbols). This indicates that DNA/rubella priming induced memory B cells, which were recalled by the boost.

We measured the anti-SIV Gag antibody response four weeks after the boost (week 61 of the study, Figure 11). For group 4 animals primed with empty rubella vaccine, the BC-sGag2 boost represented new antigens that were not seen previously. Neither macaque made anti-Gag antibodies (Figure 11A), suggesting that the vector could not elicit a primary response in the presence of rubella antibodies. The group 1 animals were quite different: they all had antibody titers to SIV Gag that were primed six months earlier (Figure 11B). In addition, they responded strongly to the live vector boost, despite the presence of rubella antibodies. In this group, the boost consisted of novel type 3 vectors expressing Gag as a combined MPER–BC-sGag2 antigen (Figure 2) or as the complete Gag protein p27. The results suggest that the gag DNA prime and first dose of live rubella vectors elicited memory B cells specific for SIV Gag antigens. These memory B cells were strongly boosted upon re-exposure to the vectors, even in the presence of rubella antibodies. The inability of control macaques (group 4) to respond in this way showed a lack of memory B cells.

## Discussion

Live attenuated rubella vaccine has a proven record of safety and immunogenicity in humans and rhesus macaques. This small RNA virus would be an attractive viral vector, if it could present the major vaccine antigens of other viruses as well as its own. This is the first report showing that live attenuated rubella vectors replicate robustly *in vivo* while expressing SIV and HIV vaccine antigens. The new vectors infected six out of six rhesus macaques, while eliciting high-titered antibodies to the SIV Gag insert in all of them and to HIV MPER in five out of six. The anti-Gag antibody titers elicited by immunization were greater than or equal to those induced by natural infection with SIV. The antibodies to both inserts have persisted for over 9 months, and they have declined at the same rate as antibodies to rubella, which protect for life. The anti-Gag antibody response was boosted by re-exposure to the vector after six months, indicating the induction of memory B cells. Since rhesus macaques are also susceptible to SIV infection, they will provide an ideal model for testing immunogenicity of novel rubella vectors and protection against SIV or SHIV challenge.

At the start of these studies, rubella vectors faced a series of questions before they could be considered a vaccine candidate. These included: location of insertion sites, size and stability of inserts, adaptation to the live attenuated vaccine strain, replication *in vivo*, immunogenicity, and concurrent immunization with more than one vector. We previously reported that rubella vectors could grow to high titers in cell culture while expressing SIV and HIV antigens at either of two insertion sites [18,19]. Inserts at the Not I site in the nonstructural region included zGFP, SIV Gag epitopes, and HIV MPER [17,18]. These inserts were limited in size and diversity, probably because they were expressed as fusion proteins with rubella nonstructural protein P150, which performs essential viral functions.

However, when the genes were inserted in the structural region, between envelope glycoproteins E2 and E1, insert expression was uncoupled from essential viral functions. This allowed the expression of larger inserts [19]. These inserts were expressed as part of the structural polyprotein under control of the strong subgenomic promoter, leading to high-level antigen expression for a prolonged period. When we switched to the rubella vaccine strain RA27/3, the new vectors were able to express the same inserts as wild type rubella, with little or no loss in vector titer, antigen expression, or insert stability [18]. The vectors with Not I deletion did not replicate well *in vivo*. This was solved by restoring the deleted sequence, which resulted in replication of type 3 vectors in six out of six macaques. The deleted sequence is part of the “Q” domain in nonstructural protein P150 with unknown function [33]. This region may be important for interferon signaling or suppression. Vectors with the Not I deletion replicate well in Vero cells, which are incapable of interferon production, but they do not replicate in normal WI38 or BSC-1 cells (Virnik et al., manuscript in preparation). Future studies will address this phenomenon.

Live replicating rubella vectors expressing SIV Gag or HIV MPER at the structural insertion site were highly immunogenic in macaques. These vectors could contribute to vaccine potency at each stage of the immune response: by simulating acute infection and triggering innate immunity, they could initiate a stronger immune response to the inserts. Subsequently, exponential growth of the vector would expose the host to increasing doses of antigen each day. Finally, prolonged antigen expression can mimic an ongoing infection [14,15], leading to germinal center formation, which is needed for immunoglobulin class switching, somatic hypermutation to produce high-affinity antibodies, and maturation of memory B cells [34]. Unlike their non-replicating homologues, live rubella vectors elicited anti-Gag antibodies after a single dose, and the antibody titers continued to rise for four to seven weeks after immunization. These strong stimuli, lasting two weeks or more, explain how live vectors could achieve the same high titers of anti-Gag antibodies as natural SIV infection.

With some other vaccines and vectors, the immune response to SIV and HIV antigens has been short-lived and lacked memory B cells [35,36]. Transient antibody responses are considered one of the major obstacles to HIV vaccine development [1]. Using live rubella vectors, the anti-Gag and anti-MPER antibodies have persisted for over nine months. They are declining with nearly the same half-life as antibodies to rubella proteins, which protect for life. In general, persistent antibody titers are thought to depend on long-lived plasma cells, while boosting depends on memory B cells, and both are signs of germinal center function during immunization [34]. The primary immune response to these vectors included memory B cells, as shown by boosting 6 months later. The secondary response depended on successful priming, and it could overcome the inhibitory effects of rubella antibodies. Potentially, two or more doses of live rubella vectors, given several years apart, could boost and update immunity to circulating strains of HIV. In addition, the ability to prime and boost memory B cells would allow us to combine rubella vectors with other viral vectors bearing similar HIV vaccine inserts [37,38], and this will be tested.

Rubella is a small RNA virus that replicates exclusively in the cytoplasm. This location is ideal for eliciting T cell immunity, since it delivers antigens directly into the proteasomal pathway leading to antigen presentation with MHC class I [39]. Priming with DNA vaccine and boosting with the vector gave high levels of Gag specific CD8+ T cells that were comparable to natural infection (Virnik, et al., manuscript in preparation). When two rubella vectors were given simultaneously, they both replicated side by side, as shown by RT-PCR, and they elicited antibodies to both Gag and MPER inserts. Prolonged expression of MPER antigen by rubella vectors may contribute to its immunogenicity and may improve on natural infection, which elicits these antibodies in less than half of the cases [40-43].

The safety and immunogenicity of live attenuated rubella vaccine were demonstrated in rhesus macaques [23] and in children [5,6,16]. Macaques have shown no signs of disease during successful immunization with rubella vectors. The safety of rubella vectors should be comparable to rubella vaccine: if a rubella vector lost its insert, it would revert to the vaccine strain. Given the overlapping host range, rhesus macaques will provide an ideal animal model for testing rubella vectors for immunogenicity and protection against SIV or SHIV challenge. Novel vectors that demonstrate vaccine potency in macaques could be quickly translated into human vaccine design.

## Conclusions

We have completed the first successful trial of live rubella vectors in rhesus macaques. Rubella vectors replicated well in all macaques tested and they provide a potent vaccine platform for immunizing with SIV and HIV antigens. These vectors have a number of desirable properties for a vaccine candidate, including: the ability to immunize with more than one antigen at a time. The vectors have shown vaccine potency comparable to natural SIV infection. They elicited long lasting immunity and induced memory B cells that can be boosted months later. They are built on a vaccine platform with known safety and immunogenicity. Due to the overlapping host range of rubella and SIV, rhesus macaques will provide an ideal animal model for testing novel rubella vectors for immunogenicity and protection against SIV or SHIV challenge.

## Methods

### Antigens antibodies and cells

Aldrithiol-2 inactivated SHIV virions with 89.6 envelope and SIV Gag proteins were a kind gift of Drs. Larry Arthur and Jeffrey Lifson at the AIDS Vaccine Program, NCI [44]. Recombinant p55 SIV Gag protein, anti-MPER monoclonal antibody 2F5 [26] and anti-HIV polyclonal antibodies used for BC-sGag2-E2TM detection were provided by the NIH AIDS Research and Reference Reagent Program, Division of AIDS, NIAID. Well-characterized recombinant gp140 SOSIP trimers [32] were a kind gift of Dr John Moore, Weill Medical College of Cornell University (New York, NY). Monoclonal 2F5 was also provided by Dr. Hermann Katinger, Polymun Scientific (Klosterneuburg, Austria). Polyclonal goat antibodies to rubella structural proteins were purchased from Fitzgerald Industries International, Inc. (Concord, MA). Vero cells were obtained from the American Type Culture Collection (Manassas, VA).

### Construction of vectors with inserts in the structural site

The derivation of type 1 and type 2 rubella vectors were described previously [18,19]. Type 3 vectors were derived from type 1 vectors by cutting out the Not I deleted Hind III-Bgl II fragment from the plasmid DNA encoding type 1 vectors and replacing it with the corresponding intact fragment from the p10RA plasmid coding for the RA27/3 vaccine strain of rubella [20]. For these vectors, the MPER insert was followed by the transmembrane domain of HIV-1 gp41 (MPER-HIVTM vector) and the E1 signal peptide. The BC-sGag2 insert consisted of four T-cell epitopes in tandem (GY9, TE15, CM9 and ME11 of SIV mac239 Gag), followed by the transmembrane domain of E2 (BC-sGag2-E2TM vector), and the E1 signal peptide (Figure 1A). All constructs were verified by sequencing, as described previously [19]. The insert sequences are provided in Figure 2. In addition to previously described inserts, one new insert combined the MPER determinant and the BC-sGag2 determinant in tandem. Another insert consisted of full-length p27 SIV Gag, containing amino acids 135–391 of SIVmac239 Gag, followed by to the E2TM domain.

The protocols for generating infectious rubella RNA from plasmid DNA, transfecting cells, passaging virus in Vero cells, viral stock expansion, sequencing the inserts, determining viral RNA content of rubella vector stocks and their titers were the same as described previously [17,18]. Briefly, we transcribed plasmid DNA *in vitro* to produce capped infectious rubella RNA, using RiboMAX Large scale RNA Production System with Ribo m7G(5’)ppp(5’)G cap analog (Promega Corp., Madison, WI). To generate rubella virus, the RNA was transfected into Vero cells, using DMRIEC reagent (Invitrogen Corporation, Carlsbad, CA). After 6–9 days of infection at 37°C, culture supernatant containing virus (passage P_0_) was collected. Infected cells were transferred onto fresh Vero cells to start a new passage. After several passages, passaging was done with cell-free culture supernatant. To produce virus stock, virus production was scaled up in T75 flasks after 5–6 passages. The supernatant was collected, titered by quantitative RT-PCR and the vector insert was sequenced. Purification of viral RNA from viral supernatants and reverse transcription were performed using QIAamp UltraSens Virus kit (QIAGEN) and High Capacity RNA-to-cDNA Kit (Applied Biosystems), respectively. The cDNA was PCR amplified with illustra PuReTaq Ready-To-Go PCR beads (GE Healthcare) and primers specific for rubella sequences flanking an insertion site, then purified in agarose gel and sequenced using the same primers.

### Detection of rubella growth in cell culture and insert expression by Western blot

Rubella structural proteins were detected by Western blot with goat anti-rubella antibodies specific for capsid protein C and envelope protein E1, as described previously [17,18]. Expression of the MPER-HIVTM and BC-sGag2-E2TM inserts was detected with human monoclonal antibody 2F5 at 1 μg/ml and anti-HIV polyclonal antibodies at 1:2500 dilution, respectively. The second antibody was either horseradish peroxidase-conjugated rabbit anti-goat IgG or goat anti-human IgG at 1:5000 dilution (Santa Cruz Biotechnology, CA). Blots were visualized with enhanced chemiluminescence (GE Healthcare).

### Animals and immunizations

Rhesus macaques, between 3 and 16 years of age were obtained from the CBER NHP colony on the NIH campus. All but two were of Indian origin; the two of unknown origin were V200 and V584. The CBER animal research program is fully accredited by the Association for Assessment and Accreditation of Laboratory Animal Care International (AAALAC). All experimental procedures were approved by the CBER Institutional Animal Care and Use Committee and were done in compliance with the current edition of the *Guide for the Care and Use of Laboratory Animals*. To be selected for the study, all animals passed a screening complete physical exam and a veterinary evaluation of their medical history including serology. Animals were negative for antibodies to rubella, herpes B, STLV-1, SIV, SRV (type D, serology/PCR) 1, 2 and 3. During the study period, the animals were negative for *Shigella*, *Salmonella*, *Yersinia*, *Campylobacter* and tuberculosis (TB) and free of intestinal pathogenic parasites.

Depending on their size, the macaques were housed in 6–12 sq ft cages and were habituated for more than 3 months. Before entry into the study, all macaques were weighed and tissue typed. They were confirmed seronegative for SIV Gag and rubella antibodies prior to receiving the vectors. All animals received ketamine anesthesia (10 mg/kg/im) for restraint during immunization, bleeding and taking oral swabs.

The 12 macaques were divided into three vaccine groups of three to four each and a control group of two animals (Figure 3). Group 3 was immunized first, with a series of three different rubella vectors, until they showed signs of a vaccine “take”, followed by two doses of DNA vaccine. Group 1 received three doses of DNA vaccine first, followed by one dose of type 3 vectors, the same ones that gave a “take” in group 3. Group 2 received three doses of DNA vaccine and was reserved for future studies. Immunizations were at weeks 0, 5, 11, 18, 25, and 31. Two animals in group 4 received the licensed rubella vaccine, which served as a vector control without an insert, followed by control DNA.

For group 1 macaques, the first two doses of DNA vaccine consisted of four plasmid DNAs: 1 mg DNA coding for SIV gag, 1 mg env DNA (gp140 and gp160), and 200 μg coding for monkey IL-12 [45]. The first dose had env DNA of clade B, while the second dose had clade C env DNA, and the third dose had equal parts of env DNA of both the B and C clades. For the animals in group 3, both DNA boosts contained env DNA of the B and C clades. All DNAs were given by intramuscular injection followed by *in vivo* electroporation using the Elgen 1000 device (Inovio Pharmaceuticals, Inc., Blue Bell, PA).

Each rubella dose consisted of a pair of rubella vectors of the same type, expressing HIV MPER and SIV Gag determinants. For group 3, the first rubella dose consisted of type 1 vectors, given IM at a dose of 10,000 PFU, followed by a second dose of 30,000 PFU. The next dose was a type 2 vector at 50,000 PFU. Finally, the type 3 vectors were given IM at a dose of 50,000 PFU. Group 1 animals received three doses of DNA vaccine, followed by a single dose of type 3 vectors at 50,000 PFU. This corresponds to about ten times the typical human dose of rubella vaccine.

Blood samples were taken before each vaccine dose and one, two and six weeks after the dose. Mouth swabs were also taken before each dose of rubella vector and one and two weeks later. The blood samples were analyzed by ELISA for antibodies to rubella, SIV Gag and HIV MPER. They were also analyzed by tetramer staining for T cells specific for the CM9 epitope. Mouth swabs were analyzed by RT-PCR using primers specific for the gag and MPER inserts.

### Detection of rubella virus in oral fluid specimens from rhesus macaques

To determine whether rubella virus inoculated in the quadriceps muscle could propagate throughout the body, we used a reverse transcription semi-nested PCR method to detect rubella sequences in oral fluid specimens from immunized rhesus macaques. The oral fluid specimens were obtained as gingival mouth swabs, using cotton swabs prior to each dose of vectors, and again one and two weeks later, using the procedure recommended by CDC [46,47]. After the collection, the sample swabs were immersed in TRIzol LS reagent (Ambion/Life Technologies, Carlsbad, CA) to preserve viral RNA, stored frozen at −80°C or promptly processed. RNA isolation was done according to the manufacturer’s protocol except for RNA precipitation step. Final steps of RNA isolation were done using RNeasy Mini Kit (QIAGEN, Valencia, CA) following the manufacturer’s RNA cleanup protocol. The final volume of elution was 60 μl. Reverse transcription was performed using ThermoScript RT-PCR System (Invitrogen/Life Technologies, Grand Island, NY) with 9 μl of the isolated viral RNA and gene-specific primers Robo-seq25, Robo-seqSbfDn4. The nested PCR was a two-step PCR using cDNA from the previous RT step and two outer primers Robo-seq25 and Robo-seqSbfDn2 complimentary to the flanking regions of the antigen inserts in the first step PCR. The cDNA was amplified for 10 cycles (20 s at 95°C, 20 s at 65°C and 40 s at 72°C) and then for 20 cycles at a different temperature profile (20 s at 95°C, 20 s at 61°C and 40 s at 72°C). The second step PCR was done with one primer specific to the insert (MPERF-seq1 for MPER-HIVTM and Robo-K241 for BC-SGAG2-E2TM) and Robo-seqSbfDn2 primer from the first step, using 1/50 of the first PCR product for 35 cycles (20 s at 95°C, 20 s at 60°C and 40 s at 72°C). PCR amplification was performed using a puReTaq Ready-To-Go PCR kit (GE Healthcare, Waukesha, WI). The products from the second PCR reaction were resolved by electrophoresis in 2% agarose gel. Sizes of the specific PCR products after the second PCR step were expected to be 347 bp for MPER-HIVTM insert and 494 bp for BC-SGAG2-E2TM insert. A positive control consisted of a plasmid coding for each insert and flanking parts of rubella. Primer sequences are given in Table 2.

**Table 2:** PCR primers used in detection of rubella virus in oral fluid specimens

**Primer**	**Sequence**
Robo-seq25	5′-cgaactggtgagccccatgg
Robo-seqSbfDn2	5′-cacaatcttggactcaaagcggac
Robo-seqSbfDn4	5′-gatctcgcaaatgcaggctccagtg
MPERF-seq1	5′-cctaggcaagaaaagaatgaaaaagaattattgg
Robo-K241	5′-tggtacgtcctagggtgcccaccggcagcgagaa

### Serum enzyme-linked immunosorbent assay

Monkey antibodies to rubella were detected by ELISA in duplicate wells, as measured on plates coated with rubella antigens (BioCheck, Inc., Foster City, CA) at the recommended serum dilution of 1:40. Macaque antibodies to SIV Gag were detected on plates coated with recombinant p55 Gag protein. For this assay, soft plastic plates were coated with 1 μg/ml recombinant p55 SIV Gag overnight at 4°. They were blocked for 5 minutes with BSA (10 mg/ml). The antibodies were then titered on the plates in PBS with 0.1 mg/ml BSA and 0.05% Triton X100. Macaque antibodies to HIV MPER were detected on plates coated overnight at 4° with recombinant gp140 SOSIP trimers at 1 ug/ml.

Macaque antibodies to MPER peptides were titered on plates coated overnight with two peptides of 14 to 16 amino acids containing the 2F5 and 4E10 epitopes. For each antigen, the plates were incubated with antibodies for 1 hour at 37°, washed twice with the above buffer, and developed with goat anti-monkey IgG conjugated to horseradish peroxidase at a 1:5000 dilution (Santa Cruz Biotechnology, CA). After 30 minutes at 37°, the plates were washed four times and developed with TMB substrate (SureBlue, KPL, Gaithersburg, MD). After ten minutes at room temperature, the reaction was stopped with 1M HCl, and OD_450_ was determined in an ELISA plate reader (Thermo Scientific).

## Competing interests

The authors declare that they have no competing interests.

## Authors’ contributions

KV designed the vectors and experiments, performed experiments, helped coordinating the study wrote the manuscript. MH planned and performed experiments and helped with revision of the manuscript. YN performed experiments and helped with revision of the manuscript. JB helped design the study, performed animal procedures and helped with revision of the manuscript. GNP and BKF helped designing and coordinating the study, provided DNA vaccine, helped with writing of the manuscript. IB designed the study and experiments, performed experiments, wrote the manuscript, coordinated and supervised the study. All authors read and approved the final manuscript.
